# β-Arrestin Promotes Wnt-induced Low Density Lipoprotein Receptor-related Protein 6 (Lrp6) Phosphorylation via Increased Membrane Recruitment of Amer1 Protein*[Fn FN2]

**DOI:** 10.1074/jbc.M113.498444

**Published:** 2013-11-21

**Authors:** Vítězslav Kříž, Vendula Pospíchalová, Jan Mašek, Michaela Brita Christina Kilander, Josef Slavík, Kristina Tanneberger, Gunnar Schulte, Miroslav Machala, Alois Kozubík, Juergen Behrens, Vítězslav Bryja

**Affiliations:** From the ‡ Faculty of Science, Institute of Experimental Biology, Masaryk University, 611 37 Brno, Czech Republic,; the § Department of Cytokinetics, Institute of Biophysics, Academy of Science of the Czech Republic, 612 65 Brno, Czech Republic,; the ‡‡ Nikolaus-Fiebiger-Center, University of Erlangen-Nürnberg, 91054 Erlangen, Germany,; the ‖ Department of Physiology and Pharmacology, Karolinska Institutet, 171 77 Stockholm, Sweden,; the ** Department of Toxicology, Pharmacology, and Immunotherapy, Veterinary Research Institute, 621 00 Brno, Czech Republic, and; the ¶ Institute of Molecular Genetics, Academy of Science of the Czech Republic, 142 20 Prague, Czech Republic

**Keywords:** β-Catenin, Membrane Lipids, Phosphatidylinositol Kinase, Phosphatidylinositol Signaling, Wnt Signaling, Amer1/WTX/FAM123B, β-Arrestin, Dvl, Lrp6 Phosphorylation, Phosphatidylinositol Phosphate Kinase

## Abstract

β-Arrestin is a scaffold protein that regulates signal transduction by seven transmembrane-spanning receptors. Among other functions it is also critically required for Wnt/β-catenin signal transduction. In the present study we provide for the first time a mechanistic basis for the β-arrestin function in Wnt/β-catenin signaling. We demonstrate that β-arrestin is required for efficient Wnt3a-induced Lrp6 phosphorylation, a key event in downstream signaling. β-Arrestin regulates Lrp6 phosphorylation via a novel interaction with phosphatidylinositol 4,5-bisphosphate (PtdIns(4,5)P_2_)-binding protein Amer1/WTX/Fam123b. Amer1 has been shown very recently to bridge Wnt-induced and Dishevelled-associated PtdIns(4,5)P_2_ production to the phosphorylation of Lrp6. Using fluorescence recovery after photobleaching we show here that β-arrestin is required for the Wnt3a-induced Amer1 membrane dynamics and downstream signaling. Finally, we show that β-arrestin interacts with PtdIns kinases PI4KIIα and PIP5KIβ. Importantly, cells lacking β-arrestin showed higher steady-state levels of the relevant PtdInsP and were unable to increase levels of these PtdInsP in response to Wnt3a. In summary, our data show that β-arrestins regulate Wnt3a-induced Lrp6 phosphorylation by the regulation of the membrane dynamics of Amer1. We propose that β-arrestins via their scaffolding function facilitate Amer1 interaction with PtdIns(4,5)P_2_, which is produced locally upon Wnt3a stimulation by β-arrestin- and Dishevelled-associated kinases.

## Teaser

**Background:** β-Arrestins are required for Wnt/β-catenin signaling, but the mechanism of their action is unclear.

**Results:** β-Arrestins bind PtdInsP_2_-interacting protein Amer1, bind PtdInsP_2_-producing kinases, and control Wnt3a-induced PtdInsP_2_ production and Amer1 membrane dynamics.

**Conclusion:** β-Arrestins bridge Wnt3a-induced Dvl-associated PtdInsP_2_ production to the phosphorylation of Lrp6 via control of Amer1 dynamics.

**Significance:** The first mechanistic explanation how β-arrestin regulates Wnt/β-catenin signaling is provided.

## Introduction

Wnt/β-catenin signaling plays a key role in homeostasis and embryonic development. Deregulation of Wnt/β-catenin pathway has been shown to cause pathophysiological conditions such as developmental abnormalities, tumorigenesis, or osteoporosis (1, 2). The Wnt/β-catenin cascade is activated by Wnts, which act as agonists for the class Frizzled (Fzd)^6^ receptors (3). When extracellular Wnt is present, it links its receptor Fzd and its co-receptor low density lipoprotein receptor-related protein 5 or 6 (Lrp5/6). Fzds bind Dishevelled (Dvl), which is required for the Wnt-induced phosphorylation of the intracellular domain (ICD) of Lrp5/6 (4, 5). Dvl and two Dvl-associated kinases, phosphatidylinositol 4-kinase type IIα (PI4KIIα) and phosphatidylinositol-4-phosphate 5-kinase type Iβ (PIP5KIβ), which produce phosphatidylinositol 4,5-bisphosphate (PtdIns(4,5)P_2_) in two sequential steps from phosphatidylinositol (PtdIns), were found to be crucial for Lrp5/6 phosphorylation (6). PtdIns(4,5)P_2_ is recognized by Amer1/WTX, which links PtdIns(4,5)P_2_ production with the machinery phosphorylating Lrp5/6 (7). Phosphorylated Lrp5/6 subsequently recruits Axin1 (8), which is a key component of the β-catenin destruction complex formed by the scaffolding proteins Axin1 and Apc (adenomatous polyposis coli) and kinases GSK3β (glycogen synthase kinase 3β) and CK1α (casein kinase 1α). As a consequence, phosphorylation and subsequent proteosomal degradation of β-catenin are blocked, β-catenin is stabilized in the cytoplasm, and following translocation into the nucleus it drives transcription of Wnt-responsive genes in cooperation with the Tcf/Lef transcription factors (9–11).

Previous studies by us and others have demonstrated the key role of β-arrestin (β-arr) scaffolding protein in Wnt signaling (for review, see Ref. 12). β-Arrestins were shown to be required for Wnt/β-catenin signaling (13, 14) as well as for branches of Wnt signaling, which do not activate β-catenin (collectively referred to as noncanonical Wnt pathways) (15–18). Whereas in noncanonical Wnt signaling β-arrestins regulate signal propagation via their well defined role in the clathrin-mediated receptor endocytosis (17, 18), the mechanism how β-arrestins control Wnt/β-catenin signaling, which does not depend on the clathrin-mediated endocytosis (19), is unclear.

In the present study we aimed to elucidate the role of β-arrestins in the process of the Wnt/β-catenin pathway activation. We demonstrate that β-arrestin is a novel binding partner of Amer1/WTX/Fam123b (further referred to as Amer1). We show that β-arrestin binds PtdIns(4,5)P_2_-producing kinases PI4KIIα/PIP5KIβ and that it is required for PtdIns(4,5)P_2_-controlled membrane dynamics of Amer1 upon Wnt3a stimulation. This function of β-arrestin governs Wnt-induced Lrp6 phosphorylation. We propose that β-arrestins acts as a scaffold, which brings Amer1 physically close to the site of Wnt3a-induced PtdIns(4,5)P_2_ production.

## EXPERIMENTAL PROCEDURES

### 

#### 

##### Cell Culture and Transfection

HEK293T cells were cultured in DMEM supplemented with 10% FBS, 2 mm l-glutamine, 50 units/ml penicillin and 50 units/ml streptomycin. Cells were transfected with polyethyleneimine (PEI) as described earlier (20). Briefly, working stocks of 25-kDa PEI were prepared from commercially available PEI (40.872-7; Sigma-Aldrich) by dilution 1:500 in PBS, pH was adjusted to 7.0, and sterile stocks of PEI were stored for months in 4 °C. DNA and stock PEI (1 μg:2.5 μl) were mixed in serum- and antibiotic-free DMEM and incubated for 30 min. The transfection mixture was then added dropwise to cells. Media were changed 3 h after transfection. Cells were harvested 24–48 h after transfection. For immunofluorescence experiments cells were transfected using calcium phosphate method or using Lipofectamine 2000 (Invitrogen).

siRNA transfection was performed on 24-well plates. Each transfection reaction contained 1 μl of Lipofectamine RNAiMAX (Invitrogen), 30 μm siRNA, and 48 μl of serum-free medium. Following the incubation for 20–30 min at room temperature the mixture was added to the suspension of trypsinized cells (0.5 ml/well). The media were changed 6 h after transfection. Cells were harvested 48 h after transfection.

Both wild type and β-arrestin1/2 DKO MEFs (21) were cultured in DMEM supplemented with 10% FBS, 2 mm l-glutamine, 50 units/ml penicillin, and 50 units/ml streptomycin. The NB4 cell line was cultured in RPMI 1640 medium supplemented with 10% heat-inactivated FBS, 2 mm l-glutamine, 50 units/ml penicillin, and 50 units/ml streptomycin.

##### Plasmids, Antibodies, and siRNA

Plasmids encoding FLAG-Amer1, EGFP-Amer1, EGFP-Amer1 deletion mutants, EGFP-Lrp6-ICD-Amer1 (7, 22), HA-β-arrestin2, FLAG-β-arrestin2, FLAG-β-arrestin2 deletion mutants (14), FLAG-hDvl3 (23), FLAG-hDvll1 (24), MYC-mDvl2 (25), EGFP-mDvl2 (16), HA-PI4KIIα and HA-PIP5KIβ (6), SuperTOPFLASH and SuperFOPFLASH Tcf/Lef (26) reporters were described earlier. *Renilla* luciferase (pRL-TK) was purchased from Promega. pEGFP-Amer1(2–838)-Lrp6-ICD was cloned by inserting Lrp6-ICD (cut from the pEGFP-Amer1-Lrp6-ICD by NotI) into a NotI site located between EGFP and Amer1(2–838) in the pEGFP-Amer1(2–832) construct. Lef1-VP16 was kindly provided by V. Kořínek (IMG AS CR, Prague) and myristoyl-mCherry by Jyrki Kukkonen.

The following antibodies were used: mouse anti-FLAG antibody (F1804; Sigma-Aldrich), rat anti-GFP antibody (3H9; Chromotek), mouse HA.11 (MMS-101R; Covance), rabbit anti-HA (ab9110; Abcam), anti-Dvl3 (sc-8027; Santa Cruz Biotechnology), anti-p(S1490)-Lrp6 (2568; Cell Signaling), anti-β-catenin (610153; BD Biosciences), anti-β-actin (sc-1615; Santa Cruz Biotechnology), anti-Myc antibody (sc-40; Santa Cruz Biotechnology), anti-α-catenin (sc-7894; Santa Cruz Biotechnology) mouse anti-Amer1 (27), rabbit anti-Amer1 (AP17838PU-N; Tocris), rabbit anti-β-arrestin (3857; Cell Signaling), anti-β-arrestin (A1CT and A2CT), a kind gift from R. J. Lefkowitz), rabbit anti-PI4KII (a kind gift from P. De Camilli), and rabbit anti-IgG control antibody (3900; Cell Signaling). siRNA sequences targeting β-arrestin1/2 (15) and PI4KII (6) were described earlier.

##### Fluorescence Recovery after Photobleaching (FRAP)

Thirty-five-mm glass-bottom dishes (MatTek) were precoated with 0.1% collagen. MEFs were transfected in suspension with 1.6 μg of EGFP-Amer1 or EGFP-Amer1(2–838) plasmid using DreamFect Gold (OZ Bioscience) according to the protocol recommended by the supplier and plated on the dish. FRAP analysis was carried out 24 h after transfection as described earlier (7) on a Zeiss LSM710 scanning microscope.

##### Immunoprecipitation and Western Blotting

Immunoprecipitation of overexpressed proteins in HEK293T cells was performed at 4 °C as described previously (28). Briefly, confluent 10-cm dishes were lysed and scraped 24 h after transfection in 1 ml of Nonidet P-40 lysis buffer (50 mm Tris, pH 7.4, 150 mm NaCl, 1 mm EDTA, 0.5% Nonidet P-40) supplied with 0.1 mm DTT and 1× Complete protease inhibitor mix (11836145001; Roche Applied Science). Lysates were spun down (16,000 × *g*, 10 min, 4 °C). Similarly, protein extracts were prepared from T75 flasks of NB4 cells for immunoprecipitation of endogenous proteins. The total protein concentration was determined using DC^TM^ Protein Assay (500-0112; Bio-Rad). Sixty μg were used as total cell lysate (TCL), 1 mg of the lysate was mixed with 1 μg of the immunoprecipitating antibody (anti-FLAG (F1804; Sigma), anti-GFP (20R-GR011; Fitzgerald), anti-HA (ab9110; Abcam), rabbit anti-β-arrestin (3857; Cell Signaling), anti-β-arrestin (A1CT and A2CT), or IgG control antibody (3900; Cell Signaling)) and kept rotating on a carousel at 4 °C. Fifteen μl of solid protein G-Sepharose beads (17-0618-05; GE Healthcare) were added to each reaction 30 min after the addition of antibodies. Test tubes were placed on the carousel overnight at 4 °C. The next day, beads were collected by spinning down at 100 × *g* for 1 min at 4 °C and washed six times with the lysis buffer. Immunoprecipitation and TCL samples were mixed with denaturing reducing Laemmli buffer, boiled, and if necessary also sonicated before loading on the SDS-PAGE. The proteins were separated according their molecular mass on 8–15% SDS-PAGE and transferred to an Immobilon-P membrane (Millipore). When required Western blots were quantified by densitometry analysis using ImageJ software.

##### Immunofluorescence Microscopy

Cell were transfected a day after plating on 0.1% collagen-precoated coverslips. Twenty four h after the transfection, cells were washed with PBS and fixed with 4% formaldehyde in PBS. Cells were washed in PBS and blocked in PBTA (1× PBS, 5% BSA, 0.25% Triton X-100, 0.01% NaN_3_) and incubated with the primary antibody overnight (4 °C). Cells were then washed three times with PBST (1× PBS, 0.1% Triton X-100) and incubated for 1 h with the secondary antibody conjugated with Alexa Fluor dye 594 or 647 (Invitrogen). Cells were washed three times with PBST, incubated with DAPI (1 μg/ml in PBST) for 10 min, and washed once in PBST. PBST was replaced with PBS, and glass coverslips were mounted with glycerol gelatin mix (079K6006; Sigma) and stored in the dark at 4 °C until scanning. Microscopy was performed on a Zeiss LSM710 laser scanning microscope or sp5 confocal microscope (Leica). Quantification of co-localization is shown as a graph of the overlap of fluorescence intensity peaks of individual channels along profiles indicated in the merged micrographs (Zen software - Zeiss in Fig. 2 and LAS AF software (Leica) in Fig. 6).

##### Dual Luciferase Assay

Dual luciferase assay was carried out in HEK293T cells. Cells were transfected on 24-well plates. Transfected cells were analyzed 24 (plasmid DNA) or 48–72 (siRNA) h after transfection according to a slightly modified protocol recommended by the supplier (E1960; Promega). Briefly, culture medium was removed, and cells were washed with PBS. Each well was lysed for 15 min at room temperature in 50 μl of lysis buffer. To measure firefly luciferase activity 20 μl from each well was pipetted into a microtiter plate and mixed with 25 μl of luciferase substrate. Luminescence was immediately measured with a Microtiter Plate Luminometer. The signal was normalized to *Renilla* luciferase activity, which was measured after addition of 25 μl of Stop and Glo mix. Graphs represent averages ± S.E. of fold changes in relative luciferase units (ratio luciferase/*Renilla*) over the control condition.

##### Membrane Fractionation

Membrane fractions were prepared from MEFs using a ProteoJET Membrane Protein Extraction kit (K0321; Fermentas) down-scaled for 6-well plate format. Briefly, cells were stimulated with Wnt3a (50 ng/ml), or alternatively the cells were treated only with an equal volume of 0.1% BSA in PBS used for dilution of Wnt3a. After 1 h of treatment, medium was removed and replaced with 1 ml of Cell Wash Solution. Each well was treated with 700 μl of ice-cold permeabilization buffer supplemented with 1 mm DTT, 10 mm NaF, and 1× Complete protease inhibitor mixture. Plates were slowly shaken on ice for 10 min. Cells were scraped and spun down at 16,000 × *g*, 15 min, 4 °C. Supernatant was used for membrane fractionation. Pellet was mixed with 60 μl of Membrane Protein Extraction Buffer and vortexed at 1200 rpm, 30 min, 4 °C. Tube content was spun down at 16,000 × *g*, 15 min, 4 °C. The membrane fraction was present in the supernatant.

Membrane and cytoplasmic fractions of NB4 cells were prepared using Subcellular Protein Fractionation kit for Cultured Cells (78840; Thermo Scientific) according to the manufacturer's recommendations. In short, NB4 cells were stimulated with Wnt3a conditioned (or control) media produced in L-cells for 2 h, spun down at 200 × *g*, 5 min, 4 °C, washed in ice-cold PBS and Wash cells by suspending the cell pellet with ice-cold PBS. One ml of ice-cold cytoplasm extraction buffer containing Complete protease inhibitor mixture was added to the cell pellet, and the tube was incubated at 4 °C for 10 min with gentle mixing. The supernatant cytoplasmic extract was gathered after centrifugation at 500 × *g* for 5 min. Next, ice-cold membrane extraction buffer containing Complete protease inhibitor mixture was added to the pellet. The tube was vortexed for 5 s on the highest setting and incubated at 4 °C for 10 min with gentle mixing. The supernatant (membrane extract) was collected by centrifugation at 3000 × *g* for 5 min. The total amount of protein was determined in each fraction by DC Protein Assay, and the extracts were subjected to immunoprecipitation and/or analyzed using Western blotting.

##### Phospholipid Species Analysis by Tandem Mass Spectrometry (MS)

Wild type and β-arrestin1/2 DKO MEFs seeded onto 150-mm plates were treated with Wnt3a conditioned (or control) media produced in L-cells and harvested into 3-ml ice-cold methanol 2 h later. Lipid extraction was performed by modified Bligh/Dyer extraction protocol. Briefly, the cell pellet was transferred by 3.0 ml of methanol into glass tubes (20 × 110 mm). The tubes were previously strongly sulfuric acid degreased and ethanol washed. After adding of 1.5 ml of chloroform and 50 μl of HCl (30%), the content was dispersed using probe sonicator at room temperature. The extraction process was continued by a 1-h incubation in an ice water bath. The above mentioned monophase extraction system was broken by the addition of 1.5 ml of chloroform and 2.5 ml of HCl (0.1 m). The samples were vigorously shaken for 1 min and subsequently centrifuged at 5000 × *g* for 60 min until two phases become clearly visible. The upper phase was carefully removed, and the lower organic phase was dried by nitrogen. Solvent mixture A (chloroform/methanol/water/glacial acetic acid/ammonium acetate solution 60:30:1:1:1 v/v) in a volume of 300 μl was used for reconstitution and the next mass spectrometric analysis using liquid chromatograph (Agilent 1200, Santa Clara, CA)-coupled mass spectrometer TripleQuand 6410 with electrospray ionization (Agilent).

Tandem MS conditions were as follows: (i) direct infusion to mobile phase; (ii) sample volume 100 μl, mobile phase flow rate 20 μl/min, mobile phase:solvent mixture A; (iii) electrospray ionization in positive mode, drying nitrogen flow 6 liters/min, fragmentor voltage and collision energy voltage optimized for each lipid group, neutral loss scan modes (*m*/*z* 277 for PtdIns lipid species, *m*/*z* 357 for PtdInsP lipid species, *m/z* 437 for PtdInsP_2_ lipid species). The amounts of selected PtdInsP lipid species, based on corresponding signal peak area, were normalized to the total PtdInsP amount in the sample.

## RESULTS

### 

#### 

##### Amer1 Is a Novel Interaction Partner of β-Arrestin2

**FIGURE 1.**
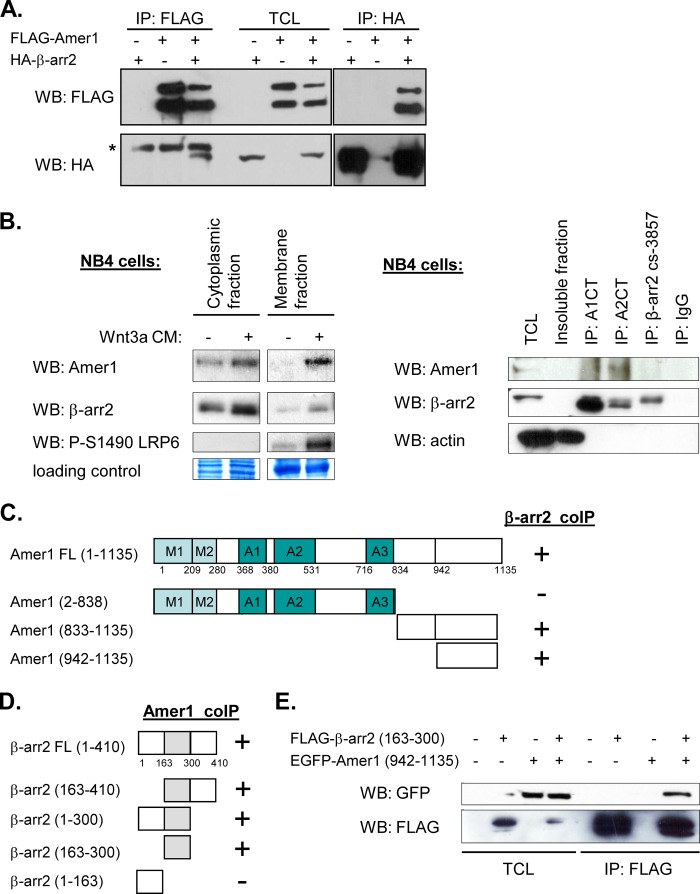
**Amer1 interacts with β-arrestin2.** *A*, HEK293T cells were transfected with FLAG-Amer1 and HA-β-arrestin2 individually or together as indicated. Cell lysates were immunoprecipitated (*IP*) with anti-FLAG or anti-HA antibody. Immunoblotting (*WB*) was done with mouse M2 anti-FLAG antibody and with mouse anti-HA antibody. TCL was used as a loading control. *B*, NB4 cells were stimulated for 2 h with Wnt3a (or control) CM, and cytoplasmic and membrane fractions were harvested. *Left panel*, NB4 cells are Wnt3a-responsive as shown by the enhanced Lrp6 phosphorylation at Ser-1490. Both Amer1 and β-arrestin2 are isolated preferentially in the cytoplasmic fraction. However, upon Wnt3a stimulation, Amer1 is recruited to the membrane and/or associates more tightly with the membrane and is therefore isolated also in the membrane fraction of NB4 cells. *Right panel*, Amer1 co-immunoprecipitated with A1CT and A2CT anti β-arrestin antibodies, but not with anti-IgG control antibody and anti-β-arrestin2 cs-3857 antibody, which poorly precipitates endogenous β-arrestin2. *C*, domain mapping of the Amer1/β-arrestin2 interaction using full-length β-arrestin2 and deletion mutants of Amer1 shows schematic view of Amer1 mutants. The interaction with β-arrestin2 is indicated with +; M1/M2 indicate regions interacting with membrane via PtdIns(4,5)P_2_; A1/A2/A3 are regions required for the interaction with Apc. Full blots are shown in supplemental Fig. 1, *A* and *B*. *D*, domain mapping of the Amer1/β-arrestin2 interaction uses full-length Amer1 and deletion mutants of β-arrestin2. The mutual interaction is indicated with +. Full blots are shown in supplemental Fig. 1*C*. *E*, the interaction between the C-terminal region of Amer1 (amino acids 942–1135) and the central part of β-arrestin2 is demonstrated by co-immunoprecipitation.

It has been shown earlier that the scaffold protein β-arrestin is a necessary component of the Wnt/β-catenin signaling pathway and a direct interactor of Dvl (13, 14). However, it is not clear how β-arrestin regulates Wnt signal transduction. To clarify this issue we have searched for high affinity physical interactions between β-arrestin and the conserved components of the Wnt signaling pathway. We have taken an advantage of the currently published study, which employed tandem affinity purification and mass spectrometry to map binding partners of 20 key components of the Wnt signaling pathway (29). Among the tested proteins only Wilms tumor-associated protein Amer1 co-precipitated with endogenous β-arrestin2. We have decided to confirm this interaction with the co-immunoprecipitation assay. As we show in Fig. 1*A*, overexpressed Amer1 and β-arrestin2 efficiently co-precipitated with each other.

Moreover, Amer1 and β-arrestin2 interaction was detected also on endogenous level in the NB4 cell line of acute promyelocytic leukemia origin (Fig. 1*B*). This cell line was chosen using the Oncomine database as a suitable candidate having high expression levels of both Amer1 and β-arrestin2. First, we checked that this cell line is Wnt3a-responsive and found that Wnt3a conditioned medium (CM) stimulates Lrp6 phosphorylation at Ser-1490, a hallmark of Wnt/β-catenin signaling (Fig. 1*B*, *left panel*). Furthermore, we detected that upon Wnt3a stimulation Amer1 gets recruited to the cytoplasmic membrane and/or associates more tightly with the cytoplasmic membrane (Fig. 1*B*, *left panel*) of NB4 cells. This protein dynamics is similar to what has been described in other cell lines (7). Endogenous immunoprecipitation in NB4 cells showed that Amer1 co-immunoprecipitated with two of three anti β-arrestin antibodies (Fig. 1*B*, *right panel*).

To define the regions of individual proteins responsible for the interaction we have performed co-immunoprecipitation assays with the EGFP-tagged deletion mutants of Amer1 and FLAG-tagged deletion mutants of β-arrestin2. These experiments revealed that it is the very C-terminal part of Amer1 (amino acids 942–1135) that is required for the interaction with β-arrestin (Fig. 1*C* and supplemental Fig. 1, *A* and *B*). On the side of β-arrestin, we can demonstrate that full-length Amer1 binds the central pocket of β-arrestin2, which is defined by amino acids 163–300 (Fig. 1*D* and supplemental Fig. 1*C*). Finally, the interaction of these two short regions of Amer1 and β-arrestin2 can be demonstrated by an efficient co-immunoprecipitation of Amer1 (amino acids 942–1135) and β-arrestin (amino acids 163–300) (Fig. 1*E*).

##### Amer1/β-Arrestin2 Co-localization in the Plasma Membrane Is Disrupted by Dvl

**FIGURE 2.**
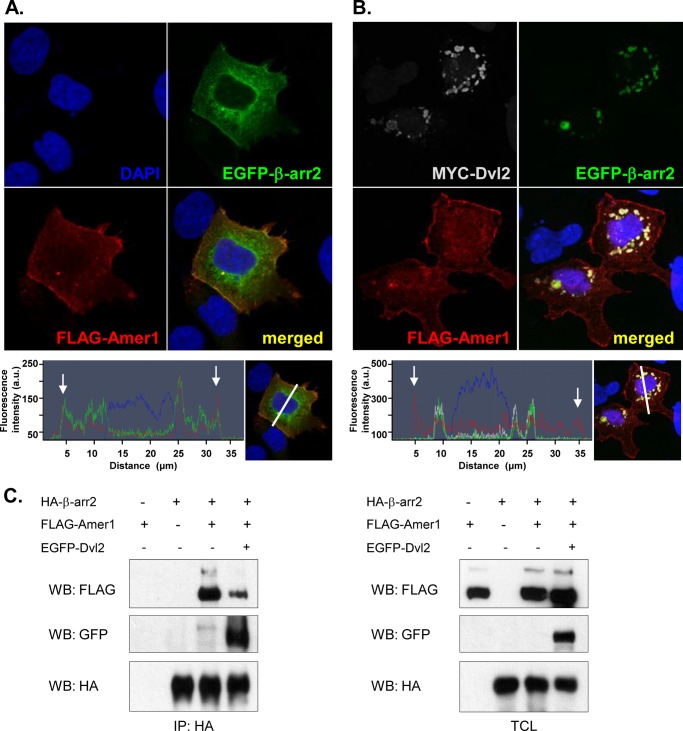
**Co-localization of Amer1 and β-arrestin2 in the proximity of cell membrane is disrupted by Dvl2.** *A* and *B*, *top*, confocal microscopy images of HEK293T cells co-transfected with the indicated plasmids, fixed, and stained with the relevant antibodies. *Bottom*, overlap of fluorescence intensity peaks along profiles indicated in the merged micrographs by a *white line. White arrowheads* indicate cell membrane position. *A*, FLAG-Amer1 (*red*) and EGFP-β-arrestin2 (*green*) co-localizing (*yellow*) at the plasma membrane. *B*, MYC-Dvl2 (*gray*) abolishing the co-localization between Amer1 (*red*)/β-arrestin2 (*green*) and recruiting β-arrestin2 to Dvl *dots. C*, HEK293T cells transfected with HA-β-arrestin2, FLAG-Amer1, and EGFP-Dvl2. The interaction of individual proteins was analyzed using co-immunoprecipitation. The efficient interaction between β-arrestin2 and Amer1 is partially abolished by Dvl2 protein.

Our findings show that Amer1 and β-arrestin2 efficiently interact with each other. In the next step we asked where in the cell this interaction takes place. We overexpressed FLAG-tagged Amer1 and EGFP-tagged β-arrestin2 in HEK293T cells and analyzed their subcellular localization by immunofluorescence. This analysis showed that Amer1 and β-arrestin2 co-localize in the proximity of the cell membrane (Fig. 2*A*). Importantly, it has been shown earlier that Dvl, another key signaling molecule in the Wnt pathway, binds, similarly to Amer1, the central part of β-arrestin2 (14). This observation opened the possibility that binding of Amer1 and Dvl to β-arrestin2 can be mutually exclusive, and β-arrestin2 is either present in complex with Amer1 or with Dvl. To test this possibility, we overexpressed Amer1, Dvl2, and β-arrestin2 and analyzed the effect of Dvl2 on the co-localization of Amer1 and β-arrestin2. As we show in Fig. 2*B* (in contrast to Fig. 2*A*), Dvl2, which is localized in the typical dots composed of Dvl2 multimers (30, 31), efficiently prevented membrane localization of β-arrestin2 and recruited β-arrestin2 to Dvl2 dots. However, the membrane localization of Amer1 was not affected. To support this observation biochemically, we have analyzed the mutual binding of Amer1 and β-arrestin2 in the presence of Dvl1, Dvl2, and Dvl3 by co-immunoprecipitation. As shown in Fig. 2*C* and supplemental Fig. 2, inclusion of Dvl3 completely and inclusion of Dvl1 and Dvl2 partially abolished the binding of Amer1 to β-arrestin2. These observations suggest that binding of Amer1 and Dvl to the central region of β-arrestin2 is competitive and that Dvl has the capacity to interfere with the interaction of Amer1 to β-arrestin2.

##### β-Arrestin Regulates Lrp6 Phosphorylation and the Activity of Lrp6-ICD-Amer1 Fusion Protein

**FIGURE 3.**
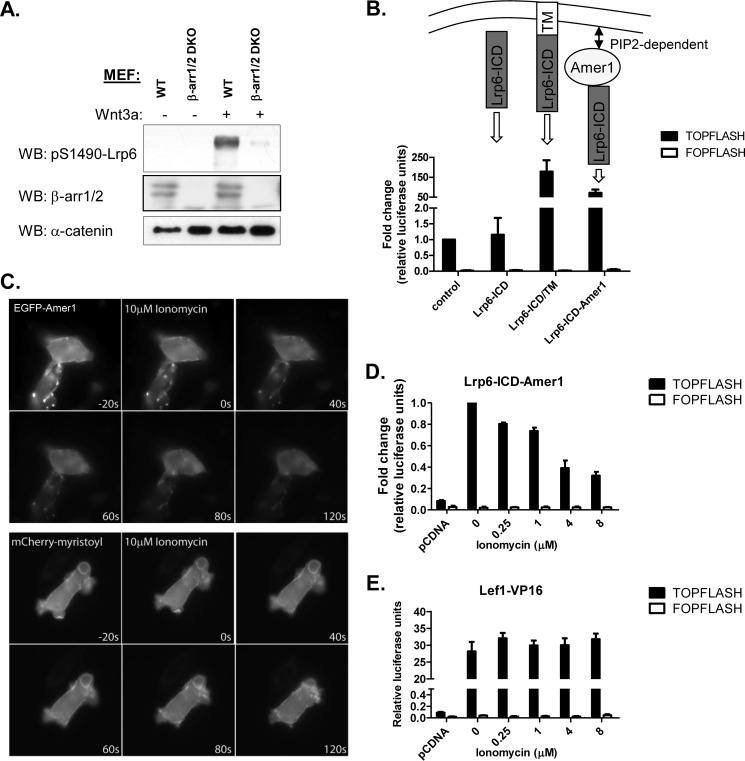
**β-Arrestin2 mediates Lrp6 phosphorylation by the regulation of Amer1 membrane recruitment.** *A*, wild type (*WT*) or β-arrestin-deficient (β-*arr1/2 DKO*) MEFs were stimulated with recombinant mouse Wnt3a (or diluent control). The analysis of phosphorylated Lrp6 (at Ser-1490) in the membrane fractions shows that the activation of Lrp6 is attenuated in β-arr1/2 DKO MEFs. α-Catenin was used as the loading control. *B*, schematic represents various Lrp6 ICD-containing constructs and their activity in the Tcf/Lef reporter assay (TOPFLASH activity). *TM*, transmembrane domain; LRP6-ICD-Amer1, fusion of Amer1 and Lrp6 ICD). The Tcf/Lef-responsive (or Wnt-responsive) construct pTOPFLASH encodes firefly luciferase reporter gene under the control of a minimal promoter and multiple copies of the optimal Tcf-binding motif CCTTTGATC. The negative control (Wnt-nonresponsive) construct pFOPFLASH harbors multiple copies of the mutant motif CCTTTGGCC. Each transfection is accompanied with constitutively expressing *Renilla* luciferase construct, which serves as an internal control for normalizing transfection efficiencies and monitoring cell viability. Graphs represent averages ± S.E. (*error bars*) of -fold changes in relative luciferase units (luciferase counts/*Renilla* counts) over the control condition. *C*, time lapse images show HEK293T cells transfected with EGFP-Amer1 or myristolated mCherry and treated with ionomycin. *Top*, EGFP-Amer1 is predominantly membranous (*t* = −20 s) but within 40 s after application of ionomycin (added at *t* = 0 s), which depletes intracellular PIP_2_ via PKC-dependent pathway, it translocates to the cytoplasm. *Bottom*, ionomycin does not alter distribution of a mCherry protein bound to membrane via its myristoyl anchor. In both experiments, GFP and mCherry photobleaching was software-controlled. *D* and *E*, HEK293T cells were transfected with the indicated plasmids and treated with ionomycin. Tcf/Lef-dependent transcriptional activity was determined by TOPFLASH reporter system. The reporter activity of Lrp6-ICD-Amer1 is dependent on PIP_2_ as demonstrated by treatment with increasing doses of ionomycin (*D*), which depletes PIP_2_ via a PKC-dependent pathway. However, ionomycin does not affect Lef1-VP16 (constitutively active form of Lef1)-driven TOPFLASH reporter expression (*E*).

It has been shown recently that Amer1 has a positive role in the Wnt3a-induced phosphorylation of Lrp6. Amer1 links Dvl-associated PtdIns(4,5)P_2_ production to the formation of Axin-GSK3β-CK1γ complexes required for the phosphorylation and activation of the Lrp6 receptor (7). Our next goal was to clarify whether or not β-arrestin has a role in this Amer1-mediated process. Importantly, the analysis of β-arr1/2 DKO MEFs demonstrates that endogenous β-arrestin is required for the efficient Lrp6 phosphorylation at Ser-1490 triggered by Wnt3a (Fig. 3*A*). These observations demonstrate that β-arrestin is required for the Lrp6 phosphorylation. In the next step we therefore tested whether or not β-arrestins regulate Lrp6 phosphorylation via regulation of the Amer1 function.

It is known that membrane targeting of Lrp6 ICD is absolutely crucial to trigger the downstream signaling by Lrp6-ICD. The cytoplasmic ICD of Lrp6 alone shows no activity (Fig. 3*B*, *first bar*) in the Tcf/Lef-dependent transcription luciferase reporter assay (TOPFLASH, (32)), which serves as a convenient readout of the activity of Wnt/β-catenin signaling in cultured cells. (In contrast when Lrp6-ICD is membrane-targeted due to the presence of the transmembrane domain (Lrp6-ICD/TM) it becomes constitutively active (Fig. 3*B*, *second bar*). Amer1 can mediate membrane targeting via its PtdIns(4,5)P_2_ binding domains, which are responsible for both Amer1 membrane recruitment and Amer1 activity in the Wnt/β-catenin pathway (7). Not surprisingly, the fusion protein of Lrp6-ICD and Amer1 (Lrp6-ICD-Amer1) (7) acted as a strong activator of TOPFLASH (but not its negative control counterpart FOPFLASH) (Fig. 3*B*, *third bar*). The activity of Lrp6-ICD-Amer1 was dependent on PtdIns(4,5)P_2_ because PtdIns(4,5)P_2_ depletion by ionomycin, which induced release of intracellular Ca^2+^ stores and subsequent cleavage of PtdIns(4,5)P_2_ by Ca^2+^ activated phospholipase C, (i) resulted in the translocation of EGFP-Amer1, but not an unrelated mCherry protein bound to membrane via a myristoyl anchor, from the membrane to the cytoplasm (Fig. 3*C*) and (ii) at the same time dose-dependently reduced the TOPFLASH reporter activity triggered by the Lrp6-ICD-Amer1 fusion protein (Fig. 3*D*), whereas the TOPFLASH activity induced by a constitutively active form of Lef1 (Lef1-VP16) remained unchained (Fig. 3*E*).

**FIGURE 4.**
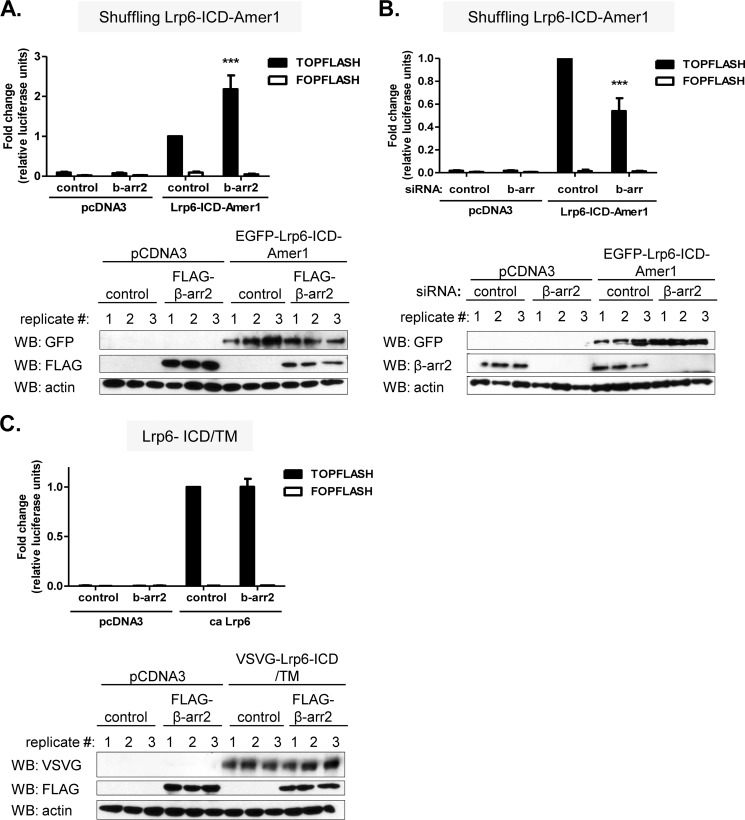
**β-Arrestin2 promotes Tcf/Lef transcriptional activity of Lrp6-ICD-Amer1 fusion protein.** *A–C*, *top*, HEK293T cells were transfected with the indicated combinations of plasmids and siRNA. Tcf/Lef-dependent transcriptional activity was determined by TOPFLASH/FOPFLASH reporter system. *Bottom*, Western blots (*WB*) of expression levels of the indicated proteins or effective knockdown or overexpression of β-arrestin are shown in each condition. Lrp6 fusion proteins are N-terminally EGFP-tagged, and β-arrestin2 is FLAG-tagged. *A*, β-arrestin2 promotes the TOPFLASH activity of Lrp6-ICD-Amer1. *B*, depletion of β-arrestin1/2 by siRNA reduces the transcriptional activity of Lrp6-ICD-Amer1. *C*, β-arrestin2 does not affect TOPFLASH activity of the constitutively active (*ca*) Lrp6 (Lrp6-ICD/TM). pcDNA3 was used as a control plasmid. Data are from at least five independent replicates. ***, *p* < 0.001 (one-way analysis of variance, Tukey's post test). *Error bars*, S.E.

Together, these experiments suggest that the activity of Lrp6-ICD-Amer1 is based on PtdIns(4,5)P_2_-dependent membrane recruitment of Amer1. This allowed us to use the Lrp6-ICD-Amer1 fusion as the readout to analyze the role of β-arrestin in PtdIns(4,5)P_2_-mediated Amer1 membrane recruitment in the context of the Wnt/β-catenin signaling. Using this system we can demonstrate that expression of β-arrestin2 promoted (Fig. 4*A*), whereas knockdown of β-arrestin1/2 decreased (Fig. 4*B*) TOPFLASH activation by Lrp6-ICD-Amer1. These observations are compatible with the possibility that β-arrestin modulates the PtdIns(4,5)P_2_-dependent membrane recruitment of Amer1. The positive effect of β-arrestin2 on the Lrp6-ICD-Amer1-induced TOPFLASH activity is not due to interference with the downstream signaling events because β-arrestin2 overexpression failed to further promote TOPFLASH activation by the constitutively active Lrp6-ICD/TM (Fig. 4*C*).

##### β-Arrestin Is Required for Wnt3a-induced Amer1 Membrane Dynamics

**FIGURE 5.**
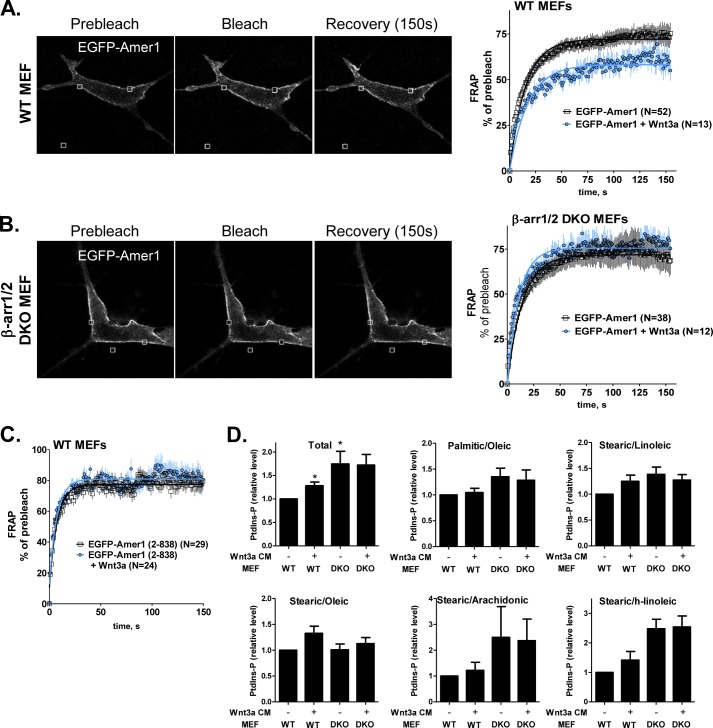
**Analysis of Amer1 membrane dynamics in the presence and absence of β-arrestin in MEF cells reveals requirement of the interaction between Amer1 and β-arrestin for Wnt3a-induced Amer1 dynamics.** *A–C*, WT and β-arr1/2 DKO MEFs were transfected with EGFP-Amer1 (*A* and *B*) or EGFP-Amer1(2–838) (*C*), and membrane dynamics of the constructs were analyzed by the FRAP method. Examples of cells subjected to FRAP are given on the *left*, *squares* indicate bleached regions. On the *right*, statistical analysis of FRAP experiment using WT and β-arr1/2 DKO MEFs stimulated with PBS or Wnt3a is shown. The graphs show mean values ± S.E. (*error bars*), and the best fitting curve model, which was used for calculation of mobile pool of EGFP-Amer1 (percentage of fluorescence recovered) and of the recovery halftime (*t*_1/2_). *N*, number of analyzed cells. Wnt3a is unable to regulate Amer1 dynamics in the absence of β-arrestin and in the case of Amer1(2–838) construct, which lacks the β-arrestin interaction domain. *D*, levels of PtdIns-P in WT and DKO MEFs stimulated with Wnt3a CM were measured by mass spectrometry. The level of selected PtdInsP was increased by Wnt3a and by the absence of β-arr. Note the lack of Wnt3a-induced dynamics in β-arr1/2 DKO MEFs. The individual lipid species from the PtdInsP pool (*Total*) are shown separately. *n* = 3. ***, *p* < 0.05 (one-way analysis of variance, Tukey's post test).

We have shown previously that Wnt3a stimulation leads to the increase in the immobilized, likely PtdIns(4,5)P_2_-bound, Amer1 fraction in the plasma membrane (7). We have applied FRAP to test whether Amer1 membrane stabilization requires β-arrestin. Wild type (WT) and β-arr1/2 DKO MEFs were transfected with Amer1-EGFP-tagged protein and subjected to FRAP essentially as described previously (7). As we show in Fig. 5, Amer1-EGFP is predominantly membranous in both WT (Fig. 5*A*) and β-arr1/2 DKO (Fig. 5*B*) fibroblasts. When we performed FRAP (exampled by two *small squares* in membrane regions and one control region outside the cell in Fig. 5, *A* and *B*) we have not observed any difference between WT and DKO fibroblast in the mobile pool of Amer1 (71.9% in WT and 72.1% in β-arr1/2 DKO MEFs) in the unstimulated conditions (Fig. 5, *A* and *B*, *right panel*). However, following Wnt3a stimulation the mobile pool of Amer1 in WT fibroblasts decreased to 58.2%, which is similar to the results, which we observed earlier in HEK293T cells (7). Interestingly, after Wnt3a stimulation β-arr1/2 DKO MEFs completely failed to regulate the mobile pool of Amer1, which only slightly increased to 75% (Fig. 5*B*, *right panel*). Moreover, the Amer1(2–838) deletion variant, which lacks the C terminus of Amer1 responsible for the interaction with β-arrestin, showed higher mobility demonstrated by a shorter recovery halftime (approximately 5 s compared with 10 s in the full-length Amer1) and a higher mobile fraction (approximately 80%) (Fig. 5*C*). Most importantly, Wnt3a stimulation was unable to change the membrane dynamics of Amer1(2–838) (Fig. 5*C*, *blue line*), which suggests that Wnt3a-triggered Amer1 membrane recruitment and/or Amer1 mobility within the membrane requires physical interaction with β-arrestin.

To further analyze the role of β-arrestin in the PtdIns phosphate metabolism, we directly measured the levels of PtdIns, PtdInsP, and PtdInsP_2_ in WT and β-arr1/2 DKO MEFs stimulated with control or Wnt-3a CM. Mass spectrometry-based analysis (for details, see “Experimental Procedures”) identified numerous PtdIns-based lipid species. Some of these species (containing the following combination of fatty acids: palmitic/oleic, stearic/linoleic, stearic/oleic, stearic/arachidonic, and stearic/h-linoleic) were detected repeatedly as PtdIns, PtdInsP, and PtdInsP_2_, which suggests that they are subject of phosphorylation by PI and PIP kinases. Interestingly, in these lipid species we have observed (i) Wnt3a-induced increase of PtdInsP and (ii) increase in the steady-state levels of PtdInsP in β-arr1/2 DKO MEFs (Fig. 5*D*). These data suggest that the defects in the dynamic Wnt3a-induced phosphorylation of PtdIns are compensated by an increase in the level of PtdInsP. Of note, lack of Wnt3a-induced dynamics accompanied by an increased steady-state amount, which is reminiscent with this phenotype, has been observed in β-arr1/2 DKO MEFs for the levels of TOPFLASH activity and of phosphorylated Dvl (14). In summary, the analysis of PtdInsPs is compatible with the possibility that the β-arr1/2 DKO MEFs are unable to respond dynamically to Wnt-3a treatment by formation of PtdInsPs and compensate for this deficit by increased steady-state levels of relevant PtdInsPs.

##### PI4KIIα and PIP5KIβ Kinases Bind β-Arr2 and Promote Amer1·β-Arr2 Complex Formation

**FIGURE 6.**
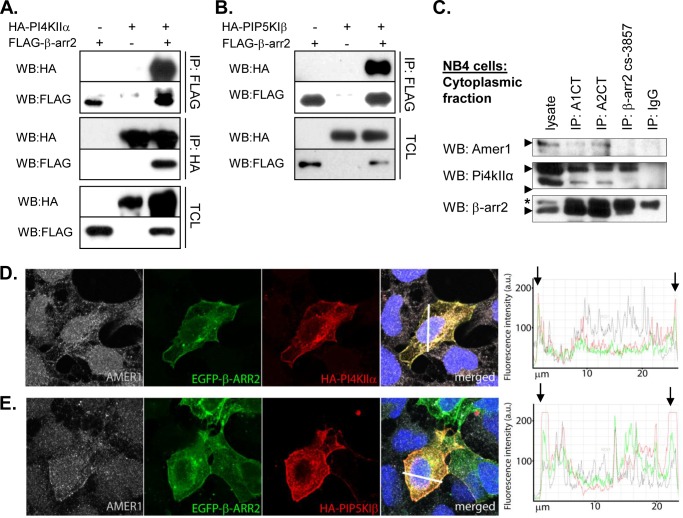
**PI4KIIα and PIP5KIβ kinases bind to β-arrestin2 and co-localize with β-arrestin2 and Amer1 in the membrane.** *A* and *B*, HEK293T cells were transfected with FLAG-β-arrestin2 and HA-PI4KIIα or HA-PIP5KIβ individually or in combination. Cell lysates were immunoprecipitated (*IP*) with anti-FLAG antibody and anti-HA antibodies, and the composition of immunoprecipitates was analyzed by Western blotting. *C*, endogenous Amer1 and PI4KIIα were co-immunoprecipitated with anti β-arrestin antibodies in the cytoplasmic fraction of NB4 cells. *D* and *E*, HEK293T cells were transfected with HA-PI4KIIα (*D*) or HA-PIP5KIβ (*E*), and EGFP-β-arrestin2 and the subcellular localization of individual proteins were analyzed by immunocytofluorescence (*left panel*). EGFP-β-arrestin2 and endogenous Amer1 (stained with rabbit anti-Amer1 antibody) co-localize with PI4KIIα/PIP5KIβ in the membrane compartment. *Right*, panels show the overlap of fluorescence intensity peaks of individual channels along profiles indicated in the merged micrographs by a *white line. Black arrowheads* indicate cell membrane position.

It has been shown that the local PtdIns(4,5)P_2_ synthesis triggered by Wnt3a is mediated by sequential action of two Dvl-associated kinases, PI4KIIα and PIP5KIβ (6, 33). Moreover, a closely related PIP5KIα was found to interact with β-arrestin2 (34). We thus hypothesized that β-arrestins, which serve as scaffold proteins, may facilitate the signal transduction by recruiting Amer1 close to the source of PtdIns(4,5)P_2_ synthesis by their interaction with PtdIns(4,5)P_2_-producing kinases. As we demonstrate in Fig. 6, *A* and *B*, both PI4KIIα and PIP5KIβ strongly interacted with β-arrestin2 physically in the co-immunoprecipitation assay. On the endogenous level, we were able to co-immunoprecipitate not only Amer1 but also PI4kIIα (Fig. 6*C*) with anti-β-arrestin antibodies in the cytoplasmic fraction of NB4 cells. Furthermore, both kinases also co-localized with β-arrestin2 and endogenous Amer1 with the most prominent co-localization observed at the membrane (Fig. 6, *D* and *E*).

**FIGURE 7.**
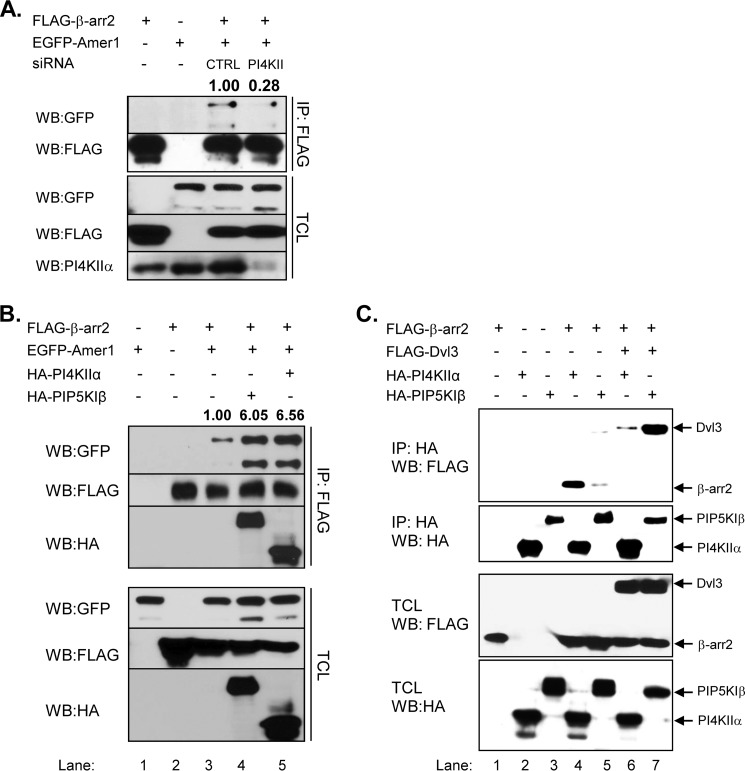
**PI4KIIα and PIP5KIβ kinases control the interaction between β-arrestin2 and Amer1.** *A–C*, HEK293T cells were transfected with the indicated plasmids and siRNAs. Interaction of individual proteins with β-arrestin2 was analyzed by anti-tag immunoprecipitation. Depletion of PI4KIIα by siRNA prevents (*A*) whereas overexpression of PI4KIIα promotes (*B*) efficient interaction of β-arrestin2 and Amer1. *Numbers* above *blots* represent the quantification of the signal by densitometry relative to the respective control condition which was set as 1. *C*, the interaction of β-arrestin2 with PI4KIIα/PIP5KIβ kinases is completely abolished by the overexpression of Dvl3.

Interestingly, when we blocked PtdIns(4,5)P_2_ production by PI4KIIα knockdown the interaction between Amer1 and β-arrestin2 significantly decreased (Fig. 7*A*). On the contrary, overexpression of either PI4KIIα or PIP5KIβ, which increases the levels of PtdIns(4,5)P_2_ (35), strongly promoted the interaction between Amer1 and β-arrestin (Fig. 7*B*). Both PI4KIIα and PIP5KIβ were found in the β-arrestin2 pulldown together with Amer1. In contrast, overexpression of Dvl3 almost completely inhibited the interaction of β-arrestin2 with PI4KIIα and PIP5KIβ (Fig. 7*C*), which is very reminiscent of the Dvl effect on the β-arrestin2·Amer1 complex (see Fig. 2). Of note, this analysis also demonstrated that β-arrestin2 has higher affinity for PI4KIIα (Fig. 7*C*, compare *third* and *fourth lanes*), whereas Dvl3 binds efficiently mainly to PIP5KIβ (Fig. 7*B*, compare *fifth* and *sixth lanes*). Taken together, these protein-protein interaction data suggest (i) that β-arrestin and Amer1 can physically interact with the PtdIns(4,5)P_2_-producing kinases PI4KIIα and PIP5KIβ, (ii) that PtdIns(4,5)P_2_ production triggers and is required for the efficient interaction of Amer1 and β-arrestin, and (iii) that the complex of Amer1·β-arr2/PI-kinase can be disrupted by Dvl3.

## DISCUSSION

In the present study we show for the first time that β-arrestins regulate Wnt3a-induced Lrp6 phosphorylation by the regulation of membrane recruitment and of the dynamics of Amer1. We propose that β-arrestin2 functionally links Fzd-associated (Fzd-Dvl-PI4KII-PIP5KI) and Lrp5/6-associated (Amer1-Axin-GSK3β-CK1γ) complexes, which are both required for the efficient downstream signaling induced by Wnt3a.

Phosphorylation of Lrp5/6 represents a key event required for downstream signaling leading to the stabilization of β-catenin and subsequent Tcf/Lef-driven transcription (8). The current model of Lrp5/6 phosphorylation pinpointed the key role of PtdIns(4,5)P_2_ as the required signal mediators, which transduce signal between Fzd/Dvl and Lrp6. Two Dvl-associated kinases, PI4KIIα and PIP5KIβ, which together in two sequential steps produce PtdIns(4,5)P_2_, were found to be crucial for Lrp5/6 phosphorylation (6). We have shown recently that the scaffold protein Amer1 (27), also known as WTX (36), which was first described as the negative regulator of the Wnt signaling (36), facilitates Wnt3a-induced Lrp6 phosphorylation (7). Amer1 is a PtdIns(4,5)P_2_-binding protein, which associates with PtdIns(4,5)P_2_ produced locally in the membrane. As a consequence of PtdIns(4,5)P_2_ production by Dvl-associated kinases Amer1 recruits Lrp6 kinases in the proximity of ICD of Lrp6 (7). Phosphorylation of Lrp6 receptors then takes place in the five-times itinerated PPP(S/T)P*X*S motives in the Lrp6 ICD. Several kinases including GSK3β, CK1γ, MAPKs, cyclin-dependent kinase, or G protein-coupled receptor kinases phosphorylating Lrp6 have been identified (37–42); however, the relative contribution of individual kinases is still a matter of debate.

**FIGURE 8.**
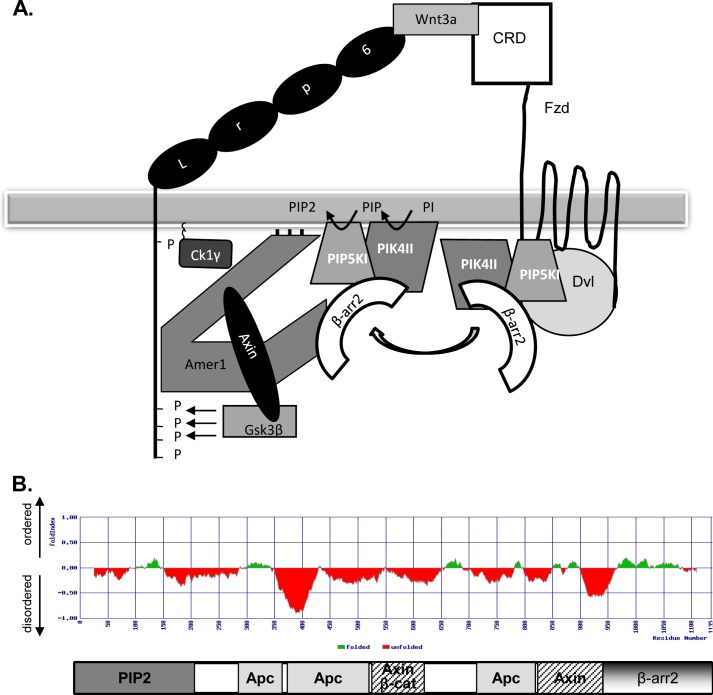
**Model: role of β-arrestin in Lrp6 phosphorylation.** *A*, the data presented in Figs. 1–7 support a model, where β-arrestin acts as a scaffold, which brings Amer1 close to the site of PIP_2_ production. A Wnt ligand activates pathway via Frizzled (*FZD*)/Dvl, which subsequently leads to the activation of PI4KIIα/PIP5KIβ kinases. We propose that the activation of Dvl and the initial production of PIP_2_ allow translocation of β-arrestin and PI4KIIα/PIP5KIβ toward Amer1-based Lrp6 phosphorylation complex composed of Amer1, Axin, and Lrp6-phosphorylating kinases CK1 and GSK3β. Local production of PIP_2_ then stabilizes the Lrp6 phosphorylation complex and feeds the phosphorylation process. *B*, the FoldIndex prediction shows that Amer1 is largely an intrinsically disordered protein (in *red*) where the domains required for binding of key proteins/metabolites required for Lrp6 phosphorylation do not overlap.

Based on our data we propose a model of β-arrestin and Amer1 function in the Wnt3a-induced phosphorylation of Lrp6 (schematized in Fig. 8*A*). The model is based on our finding that β-arrestin can be either present in complex with Dvl or with Amer1. The interaction of Amer1 and β-arrestin takes place near the membrane and most importantly requires PtdIns-P_2_ and possibly other membrane lipids or membrane itself.

The interaction of β-arrestin with Dvl seems to have higher affinity than the interaction of β-arrestin with Amer1 or PI4KII/PIP5KI kinases. As a consequence, overexpression of Dvl is able to efficiently disrupt the binding of β-arrestin with Amer1 and PI4KII/PIP5KI (see Figs. 2 and 7). It is known that the Wnt signaling cascade is induced by binding of Wnt to Fzd. This interaction subsequently triggers by an yet unidentified mechanism involving Dvl the activation of PtdIns(4,5)P_2_-producing kinases PI4KII and PIP5KI (6).

The production of PtdIns(4,5)P_2_ is required and further promotes the interaction of Amer1 and β-arrestin. We speculate that following the activation, Dvl undergoes a conformational change (induced either by posttranslational modifications or by recruitment of other proteins), which breaks the β-arrestin/Dvl interaction and allows the formation of the β-arrestin·Amer1 and β-arrestin·PI4KII·PIP5KI complexes. β-Arrestin thus acts as a switch, which translocates PtdIns(4,5)P_2_-producing kinases from Dvl toward the Lrp6-phosphorylating complex. This allows efficient phosphorylation of Lrp6 fed by the local production of PtdIns(4,5)P_2_ associated with β-arrestin. Indeed, direct measurements of PtdInsP and PtdInsP_2_ showed (i) a Wnt3a-induced increase in these PtdInsPs and (ii) an increase in the steady-state levels of PtdInsP in β-arrestin1/2 DKO MEFs. These data suggest that the defects in the dynamic Wnt-induced phosphorylation of PtdIns are compensated by increase in the level of PtdInsP. Of note, similar phenotype (lack of Wnt-induced dynamics accompanied by increased steady-state activation) has been observed in β-arrestin1/2 DKO MEFs for the levels of TOPFLASH activity and of phosphorylated Dvl (14).

The known properties of Amer1 make the scenario schematized in Fig. 8*A* sterically possible. The individual regions of Amer1, which interact with PtdIns(4,5)P_2_ (7), APC (27), Axin (22), and β-arrestin (this study) do not overlap (Fig. 8*B*). The N-terminal part of Amer1 recognizes PtdIns(4,5)P_2_ and central regions interact with Lrp6 and Axin, whereas the very C-terminal region is responsible for the interaction with β-arrestin. Moreover, Amer1 is, based on the computer predictions, an intrinsically disordered protein with the lack of clearly defined secondary structure (Fig. 8*B*). This feature allows Amer1 to act as scaffold and to exist in numerous conformations depending on the individual binding partners.

Amer1 has a dual role in Wnt/β-catenin signaling. It was first identified as a negative regulator of Wnt/β-catenin signaling, which interacts with the components of the destruction complex (Apc, Axin, GSK3β, CK1) (7, 27, 36). Amer1 was identified only recently also as a positive regulator of the Wnt/β-catenin signaling, which acts at the level of Lrp6 phosphorylation (7). It is of interest that its positive role seems to be limited to Amer1 and does not apply to related Amer2 (43), which lacks the C-terminal sequence (44) required for the interaction with β-arrestin.

In summary, in the present study we provide the so far missing molecular mechanism utilized by β-arrestin to positively regulate Wnt/β-catenin signaling. According to our data β-arrestin acts in the Wnt/β-catenin pathway via Amer1, which is a protein conserved only in vertebrates. This raises the possibility that the function of β-arrestin in the Wnt/β-catenin pathway evolved in parallel and will be limited to vertebrates. This is in agreement with the lack of Wnt/β-catenin-related phenotypes in the *Drosophila* β-arrestin homologue kurtz and the *Caenorhabditis elegans* homologue arr-1 mutants. Phosphorylation of Lrp6 via the β-arrestin/Amer1 pathway thus represents a mechanism for the efficient and tightly controlled activation of the Wnt/β-catenin pathway that evolved in vertebrates.

## Footnotes

6 The abbreviations used are:

Fzd: Frizzled
Apc: adenomatous polyposis coli
β-arr: β-arrestin
CK1: casein kinase 1
CM: conditioned medium
DKO: double knock-out
Dvl: Dishevelled
EGFP: enhanced green fluorescent protein
FRAP: fluorescence recovery after photobleaching
GSK3β: glycogen synthase kinase 3β
ICD: intracellular domain
Lrp: low density lipoprotein receptor-related protein
MEF: mouse embryonic fibroblast
PI4KIIα: phosphatidylinositol 4-kinase type IIα
PIP5KIβ: phosphatidylinositol-4-phosphate 5-kinase type Iβ
PtdIns: phosphatidylinositol
PtdIns(4,5)P_2_: phosphatidylinositol 4,5-bisphosphate
TCL: total cell lysate.
